# Investigating the impact of crisis management training on clinical decision-making and management of stress factors in the personnel of emergency medical services: randomized controlled trial

**DOI:** 10.1186/s12909-025-07730-6

**Published:** 2025-08-20

**Authors:** Ghasem Babamiri, Zohreh Karimiankakolaki

**Affiliations:** Social Determinants of Health Research Center, Shk.C., Islamic Azad University, Shahrekord, Iran

**Keywords:** Education, Crisis management, Decision making, Stress, EMS

## Abstract

**Introduction:**

Emergency medical workers face unique problems that are specific to their work environment because they play an effective role in happy and sad moments of life and death, accidents and disasters, and these people face a high level of tension. The purpose of this study is to investigate the impact of crisis management training on clinical decision-making and management of stress factors in the personnel of emergency medical services (EMS).

**Methods:**

In this randomized controlled trial with parallel groups study, 64 personnel of the emergency medical center of Shahrekord University of Medical Sciences were selected in two groups of 32 people, control and intervention in 2024. The training content included how to deal with the crisis and crisis management. 32 training sessions were held in two weeks. After the necessary training, the effectiveness of the training on the personnel in line with crisis management was checked through a questionnaire and its relationship with process improvement. Clinical decision-making was investigated. Demographic characteristics, nursing stressors of Gary Taft and Anderson and clinical decision-making questionnaire were used. The data was analyzed with spss23 software.

**Results:**

The average age of the study subjects in the intervention and control groups was 31.86 and 30.98 years. Before the intervention, there was no significant difference in the average of clinical decision making in the two groups (*p* > 0.05), but after the intervention, there was a statistically significant difference (*p* < 0.05). Also, in the intervention group, there was a significant difference in the decision-making score before and after the intervention (*p* < 0.05). In the two groups’ intervention and control, the most common type of decision-making was analytical-intuitive decision-making (71.88 and 65.62%).

**Conclusion:**

The results of the present study showed that crisis management training interventions can reduce job stress and tension in prehospital emergency personnel and improve their decision-making process.

## Introduction

A disaster is a destructive event that requires extensive emergency assistance to ensure the health and survival of the affected population. Crisis management is the process of reducing the risk of a disaster using anti-crisis resources in an efficient and effective manner [1]. Crisis management has 4 stages, each of which is designed and implemented with its own specific goals. These stages are: prediction and prevention with the aim of reducing the likelihood of a crisis; preparation with the aim of planning, training and research; coping with the aim of providing emergency services immediately after the crisis; reconstruction with the aim of returning society to normal; Crisis management, as part of its tasks as prediction and prevention, will be able to increase public awareness of the risks of natural disasters and create appropriate changes in people’s behavior by preparing appropriate educational programs [2].

Iran is one of the 10 most disaster-prone countries, with 90% of its population exposed to natural disasters [3]. Iran, with the highest number of accidents (27,000 deaths and 300,000 to 500,000 injuries), is among the countries where the conditions of pre-hospital services are similar to other developing countries. The incidence of traffic accidents in Iran is higher than the global average, with one person dying every 33 min due to traffic accidents [3].

For this reason, in recent years, risk, risk management, rescue and emergency operations have increasingly become a topic of research and discussion in the country. Emergency medical services are an important part of the health system and are considered a public good in most societies [4].

A significant portion of deaths, especially in developing countries, occur in pre-hospital settings. A study in Iran showed that prehospital, emergency department, and hospital mortality accounted for 20, 42, and 37% of trauma deaths, respectively [5]. Therefore, prehospital emergency care plays a vital role in saving human lives [6]. One of the activities in emergency care is clinical decision-making, which is the most important and at the same time the most risky part of health professions. Therefore, knowing decision-making and applying useful strategies to develop this skill in health care workers, especially emergency medical staff, is essential [7].

Because emergency medical personnel, in addition to the problems that exist for individuals in society, face unique problems specific to their work environment, such as: working with multiple staff and individuals in the treatment team, patients and families in crisis, happy and sad moments of life and death, accidents and disasters, and accidents. Therefore, these individuals face a high level of stress [8].

Emergency medical personnel, in their professional role, must make a large number of decisions daily, decisions that are related to the continuation of the patient’s life. Therefore, clinical decision-making will be a complex process [9]. Decision-making is the most fundamental and continuous component of the care system, especially in emergency situations [10].

Because emergency units are environments full of complexity and the extremely high workload, the need to use information, the sensitivity of seconds, extreme tension, unpredictability, and the criticality of diagnosing problems and waiting for companions and preserving the lives of patients distinguish these departments from other departments. Therefore, it is essential that the staff in these departments have sufficient capabilities, skills, and information to solve problems and make decisions in any situation [11, 12]. In addition, patients and their families expect these individuals to make the best decisions in dealing with their needs [13]. In particular, emergency nurses must interview patients before a physician can perform a medical examination and are therefore the first to respond to patients and their families. They are also acutely aware of the pressure to determine the level of triage in a limited time frame [14]. According to a study, poor clinical decisions by nurses are the main cause of around 34% of medical complications in British hospitals, 6% of which lead to permanent disability and 8% to patient death. It should also be noted that half of these deaths could be prevented by timely decision-making by nurses [15].

One study reported that when nurses are overexposed to stressors, the quality of nursing care decreases [16]. Since the personnel of emergency medical services have relatively low awareness of crisis management and related factors, there is a need for training and drills to increase awareness in order to cope with perceived risks [17].

A study found that emergency medical service managers should plan to improve the quality of work life of their employees, especially in terms of work and overall living space [18]. The study by Froutan et al. showed that stress management training can play an acceptable role in reducing the level of anxiety and increasing the resilience of emergency medical workers. Since reducing job stress in these individuals can lead to better clinical services, it is essential to use these effective strategies to reduce job stress and increase resilience [19].

While EMS personnel face high stress [14, 16, 20], few studies have tested how crisis training impacts their decision-making under pressure. To survive in crisis situations, organizations are forced to adopt drastic and often management-driven work practices that can cause high levels of stress, low productivity, and absenteeism among employees. Therefore, implementing more inclusive and responsive management to stressors can help reduce employee stress levels in crisis situations [21].

There is growing evidence demonstrating the effectiveness of training programs in enhancing team performance and reducing adverse patient outcomes in real-world clinical settings. As such, simulation-based training is an essential component of educational initiatives aimed at improving healthcare delivery and patient safety [22].

Given that EMS personnel must perform at their best in critical situations, they are often faced with making decisions under pressure, we hypothesized that crisis training would reduce stress and improve analytical-intuitive decision-making. Therefore, the purpose of the present study was to investigate the effect of crisis management training on clinical decision-making and management of stress factors in the personnel of emergency medical services of Shahrekord University of Medical Sciences in 2023.

## Methods

### Study design

A randomized controlled trial with parallel groups.

### Setting

The present study was an interventional study with pre-test and post-test, which was conducted at Shahrekord University of Medical Sciences and with the target population of emergency medical services.

### Sample size

The sample size required in this study was calculated based on the formula comparing two population means by conducting a pilot study and an estimate of the standard deviation (s) of stress score with a confidence interval of 95% (1.96) and a test power of 80% (0.84) and 10% probability of dropping samples, of 32 participants in each group.

### Participants

This study was conducted in 2024. The inclusion criteria for the study subjects included being employed in the emergency medical services department, not participating in crisis management training programs in the past 6 months, having at least an associate’s degree, and having at least two years of work experience in the emergency medical services department. The exclusion criteria also included dissatisfaction with continuing cooperation, not fully participating in the training program, transfer to other departments, and completion of service.

### Randomization

Participants were divided into intervention and control groups based on a computer-generated random number. The assigned groups were kept in opaque sealed envelopes. This trial was conducted in accordance with the CONSORT statement.

### Study procedure

This study was conducted on emergency medical personnel of Shahrekord University of Medical Sciences. At the beginning of the study, a briefing session was held for the participants, and the study objectives and methods were explained. Participant characteristics and written informed consent were obtained from all participants. Each participant received pre-test questionnaires and a random sealed envelope. After group division, educational intervention was provided to the intervention group, but the control group did not receive any intervention. The intervention was through holding a training course and a crisis response maneuver, and the participation of emergency medical services personnel in these courses. In general, 32 training sessions were held in line with the study objectives, and after 2 months, a post-test was administered to the participants in the study.

### Educational intervention

The training content included how to deal with crises and crisis management. The training material was divided into several sections and each section was taught on a different day. The training time was determined in coordination with the personnel. Also, conditions were provided for holding question and answer sessions to resolve problems and issues. The expected duration of the training was two weeks and 4 one-hour sessions each day. Each 1-hour session was a combination of lecture (30 min) and scenario-based simulation (30 min).

The training course was conducted by 4 emergency medical specialists and experts and the following topics were covered in the course: Crisis and concepts - related laws and programs, Crisis management-recovery cycle Status of crisis in Iran and the world Crisis risk assessment Crisis management structures Harm reduction and stress reduction measures in dealing with the injured Early warning system Management of incidents with mass casualties. In addition to crisis management topics, in lectures and Q&A sessions, the trainers also discussed stress management and self-control strategies in stressful situations. The training content was derived from the comprehensive training program in the field of crisis management from the Ministry of Health and Medical Education of Iran. In addition, in each session, the trainers mentioned concrete examples from their experiences in crisis situations, considering the topic.

The time spent on the topics sometimes differed from the suggested schedule provided by the researcher based on the needs of the classes and the audience.

**Table:** Training sessions

Meeting topic	Duration of each session	Training method	day
Crisis and concepts - related laws and programs	4 one-hour sessions	Lecture and Q&A	First day
Crisis management-recovery cycle	4 one-hour sessions	Lecture and Q&A	Second day
Status of crisis in iran and the world	4 one-hour sessions	Lecture, Q&A and Scenario-Based Simulation	Third day
Crisis risk assessment	4 one-hour sessions	Lecture, Q&A and Scenario-Based Simulation	Fourth day
Crisis management structures	4 one-hour sessions	Lecture, Q&A and Scenario-Based Simulation	Fifth day
Harm reduction and stress reduction measures in dealing with the injured	4 one-hour sessions	Lecture, Q&A and Scenario-Based Simulation	Sixth day
Early warning system	4 one-hour sessions	Lecture and Q&A and Role-Play	Seventh day
Management of incidents with mass casualties	4 one-hour sessions	Lecture and Q&A and Role-Play	Eighth day

### Assessment tools

The required information was extracted by three questionnaires. The first questionnaire is the demographic characteristics of the study subjects, which include age, gender, education level, and work experience.

The second part of the tool includes the Gary Taft and Anderson Nursing Stress Questionnaire. The Nursing Stress Questionnaire (ENSS) was designed and validated by Gary Taft and Anderson (1981) [23]. This questionnaire consists of 57 closed-ended response items based on a five-point Likert scale. The ENSS scale is a revised version of the NSS Nursing Stress Scale. The NSS is the first tool designed to measure nursing stress instead of general job stress. This questionnaire was validated by Mohammad Gholizadeh (2015). The questionnaire was scored using a Likert scale (I am not stressed at all1, I am sometimes stressed2, I am often stressed3, I am extremely stressed4, this situation does not include my duties5). If the questionnaire scores are between 57 and 114, the level of variability in this population is weak. If the scores of the questionnaire are between 114 and 228, the level of variability is at a moderate level. If the scores are above 228, the level of variability is very high. Cronbach’s α was 0.84 for the stress scale (Gholizadeh, 2015) [24].

The third part will be conducted with the Clinical Decision-Making Questionnaire. This questionnaire is a Persian translation of the Clinical Decision-Making Questionnaire by Lowry, Selantra, Chalmers et al. in Turkey, which is the result of an extensive literature review and qualitative study and consists of 32 items [25]. This questionnaire consists of 24 closed-ended questions with a 5-point Likert scale (always, rarely, moderately, often, always) that are scored from 1 to 5, respectively. Questions 1, 3, 5, 7, 9, 11, 13, 15, 17, 19, 21 and 23 are scored in reverse order. Study participants can obtain a score from 24 to 120. If the score is below 67, decision-making is of a systematic analytical type. If it is between 68 and 78, it indicates the second level of decision-making, i.e., analytical-intuitive, and if it is above 78, it indicates the third level of clinical decision-making, i.e., intuitive-interpretive. Cronbach’s α was 0.80 for the Clinical Decision-Making scale (Javadi, 2008) [26].

### Ethical Considerations

Ethical approval for this study has been obtained by the ethics committee affiliated with Islamic Azad University, Shahrekord Branch, Iran (reference number IR.IAU.FALA.REC.1402.013) and Informed consent was obtained from all study participants. In order to comply with ethical issues, all those who formed the research samples were initially provided with the necessary explanations about the purpose of the research and how the study was conducted, and their participation in the study was voluntary. In addition, sufficient considerations were taken regarding the confidentiality and privacy of the samples’ personal information, and the research samples were also reassured.

In order to comply with ethical considerations, at the end of the study, the control group was given the educational intervention.

### Statistical analysis

**Fig. 1:**
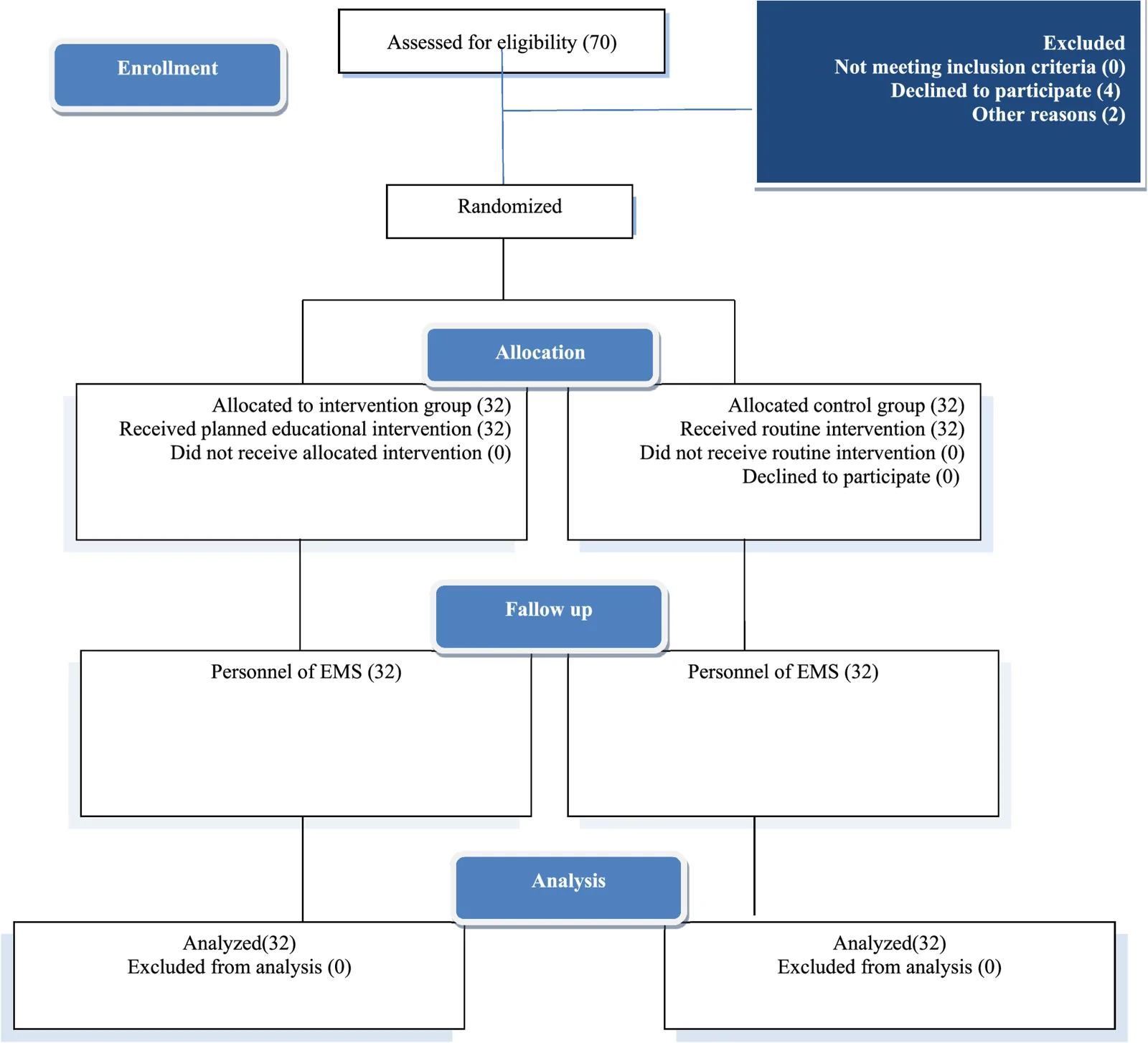
Research steps

In the present study, quantitative traits were reported as mean (standard deviation). Qualitative traits were reported as number (percentage). The normality of data distribution was assessed using the Kolmogorov-Smirnov test. The mean of quantitative traits was compared between different subgroups using independent t-tests and their ANOVA. Various relationships were examined through correlation analysis and linear regression. Complementary analyses, including effect size, were reported. SPSS version 23 software was used to analyze the data Fig. 1.

## Results

The demographic characteristics of the participants in the intervention and control groups are presented in Table 1. As is clear, none of the demographic characteristics differed significantly between the two groups. In other words, the two groups were homogeneous in terms of demographic factors. The average age of the study subjects in the intervention and control groups was 31.86 and 30.98 years, respectively. 50% of the participants in the intervention group were in the age range of 30 to 40 years. Most of the employees had an associate’s degree (59.37%). The average work experience of the individuals also varied from 5 to 10 years (Table 1).

**Table 1:** Demographic characteristics of participants (*n* = 32/group)

variable	intervention			Control		*P*-value
	*N*		%	*N*	%	
Age	3.55 ± 31.86			3.48 ± 30.98		0.125
Under 30 years	11		34.37	13	40.62	
30–40 years	16		50	15	46.87	
40 to 50 years	3		9.37	2	6.25	
Over 50 years	2		26.6	2	6.25	
Gender						
Male	30		93.75	29	90.62	0.55
Female	2		6.25	3	9.38	
Education						
Diploma or Associate Degree	19		59.37	17	53.12	0.65
Bachelor’s Degree	12		37.5	14	43.75	
Master’s Degree and Above	1		3.13	1	3.13	
Work experience						
Less than 5 years	4		12.5	5	15.63	0.11
5 to 10 years	12		37.5	15	46.87	
More than 10 years	16		50	12	37.5	

The mean and standard deviation of the clinical decision-making score of the personnel are presented in Table 2.

**Table 2:** Comparison of the mean clinical decision-making scores pre-post intervention (*n* = 32/group)

Time	Standard deviation ± mean		Independent t-test	
	intervention	control	t	*P*
Before intervention	4.26 ± 72.15	4.55 ± 71.76	0.35	0.65
After intervention	4.26 ± 80.08	4.32 ± 72.12	11.54	0.001>
Paired t-test (p-value)	0.012 Mean difference: 7.93 Cohen’s d: 1.86 [95% CI: 6.39–9.47];	0.069 Mean difference: 0.36 Cohen’s d: 0.08 [95% CI: −1.24–1.96];		

In the intervention group, the mean clinical decision-making score before and after the intervention was 80.08 ± 4.62 and 72.15 ± 4.26, respectively (Mean difference: 7.93, Cohen’s d: 1.86 [95% CI: 6.39–9.47]; *p* = 0.012). In the control group, these values ​​were 71.76 ± 4.55 and 72.12 ± 4.32 (Mean difference: 0.36 Cohen’s d: 0.08 [95% CI: −1.24–1.96]; *p* = 0.069).

In Table 2, the t-test showed that before the intervention, the mean clinical decision-making score in the intervention and control groups did not differ significantly (*P* > 0.05). However, after the intervention, the mean clinical decision-making score in the two groups differed significantly (*P* < 0.001). The paired t-test also showed that in the intervention group, the clinical decision-making score before and after the intervention was significantly different, but these changes were not significant in the control group.

As shown in Table 3, analytical-intuitive decision-making was most common (71.88%) in the intervention group and (56.62%) in the control group.

**Table 3:** Decision-making models post-intervention (*n* = 32/group)

Clinical Decision Making Model	Score Range	Number (percentage)	
		Intervention	Control
Intuitive Decision Making	Above 78	5(15.62)	21(12.50)
Analytical-Intuitive Decision Making	68–78	23(71.88)	44(56.62)
Analytical Decision Making	Less than 68	4(12.50)	7(21.88)

As shown in Table 4, in the intervention group, the mean stressors score before and after the intervention was 185.28 ± 10.32 and 136.23 ± 8.25, respectively (Mean difference: 49.05 Cohen’s d: 5.19 [95% CI: 52.46–45.64]; *p* = 0.001). In the control group, these values ​​were 181.16 ± 9.85 and 185.56 ± 11.02 (Mean difference: 4. Cohen’s d: 0.42 [95% CI: 0.63–8.17]; *p* = 0.068). The magnitude of these effect sizes is likely to be overestimated due to factors such as self-report measures, short follow-up, and small sample size, and requires cautious interpretation and future replication.

**Table 4:** Comparison of the mean stress factors scores pre-post intervention (*n* = 32/group)

Time	Standard deviation ± mean		Independent t-test	
	Intervention	Control	t	*p*-value
Before intervention	10.32 ± 185.28	9.85 ± 181.16	0.45	0.125
After intervention	8.25 ± 136.23	11.02 ± 185.56	10.09	0.001>
Paired t-test (*p*-value)	0.001 Mean difference: 49.05 Cohen’s d: 5.19 [95% CI: 45.64–52.46]	0.068 Mean difference: 4.40 Cohen’s d: 0.42 [95% CI: 0.63–8.17]		

In Table 4, the t-test showed that before the intervention, the mean stressors score in the intervention and control groups did not differ significantly (*P* > 0.05). However, after the intervention, the mean stressors score in the two groups differed significantly (*P* < 0.001). In general, the stressors score in both groups before and after the intervention was at an average level. The paired t-test also showed that in the intervention group, the stressors score before and after the intervention was significantly different, but these changes were not significant in the control group.

As shown in Table 5, among the demographic characteristics, the score of stress factors according to education and work experience has a significant difference (*p*-value < 0.05, SPSS23).

**Table 5:** The mean score of stress factors pre-post intervention according to demographic characteristics

variable	before		after		*P*-value
	mean	SD	mean	SD	
Under 30 years	186.55	10.15	180.52	11.12	0.056
30–40 years	180.12	11.25	181.21	9.10	
40 to 50 years	179.35	10.09	184.32	10.15	
Over 50 years	176.89	10.11	179.15	10.29	
Gender					
Male	183.15	9.98	185.45	9.89	0.06
Female	184.32	10.45	182.98	10.10	
Education					
Diploma or Associate Degree	183.55	11.21	180.52	11.12	0.012
Bachelor’s Degree	179.65	10.20	179.63	10.59	
Master’s Degree and Above	177.55	9.95	177.98	10.58	
Work experience					
Less than 5 years	180.25	11.15	175.78	10.52	0.003
5 to 10 years	184.32	11.14	178.65	9.21	
More than 10 years	186.02	10.13	179.65	10.09	

As shown in Table 6, among the demographic characteristics, the clinical decision-making score is significantly different according to age, education, and work experience (*P* < 0.05, SPSS23).

**Table 6:** The mean score of clinical decision-making pre-post intervention according to demographic characteristics

variable	before		after		*P*-value
	SD	SD	mean	SD	
Under 30 years	80.15	4.45	71.55	5.60	0.0045
30–40 years	80.08	4.12	72.25	4.81	
40 to 50 years	81.12	4.10	70.45	4.30	
Over 50 years	79.10	4.61	69.15	4.36	
Gender					
Male	79.80	4.45	70.14	4.62	0.085
Female	80.19	4.25	71.23	4.39	
Education					
Diploma or Associate Degree	81.10	3.95	70.78	5.12	0.002
Bachelor’s Degree	80.25	4.5	71.19	4.70	
Master’s Degree and Above	79.85	4.42	72.05	4.18	
Work experience					
Less than 5 years	81.15	4.25	70.77	5.33	0.003
5 to 10 years	82.02	4.63	69.92	4.19	
More than 10 years	79.24	4.28	68.90	4.5	

## Discussion

One of the main variables examined in the present study was determining the mean clinical decision-making score. After the intervention, the mean decision-making score in the two groups showed a statistically significant difference and was higher in the intervention group than in the control group.

In a study conducted by Beigi et al. (2015) with the aim of examining the effect of decision-making skills training on the understanding of clinical decision-making among nursing students, the results showed that the group that underwent decision-making skills training had a higher understanding of clinical decision-making [27]. The results of the study by Ghodsi Astan et al. (2022) indicate the positive effect of evidence-based education on nurses’ clinical decision-making. Therefore, it is recommended that nurses use evidence-based education methods to improve their clinical decision-making level [28]. In a study conducted by Beigi et al. (2015) with the aim of examining the effect of decision-making skills training on the understanding of clinical decision-making among nursing students, the results showed that the experimental group had a significant difference from the control group in three of the four dimensions of the Clinical Decision-Making Understanding Questionnaire and the total score of the test. In fact, the group that underwent decision-making skills training had a higher understanding of clinical decision-making [27].

Operators are always likely to change decision criteria, albeit unconsciously, to conform to their preconceived notions.

Also, in recent years, critical environments have become increasingly faster-paced and relatively more dynamic than previously thought, resulting in a lack of time for medical staff to perform complex calculations for decision-making. A study showed that half of deaths could be prevented by timely clinical decision-making by nurses [15]. Therefore, training in crisis management is an essential part of the training of EMS emergency personnel and should be planned.

In the present study, the type of decision-making (analytical-intuitive, intuitive, and analytical) was examined.

According to the results presented in Table 3, in the intervention and control groups, the most common type of decision-making was analytical-intuitive decision-making (71.88 and 65.62%). Our results align with Okoli (2018), who found analytical-intuitive decision-making dominant in crises [29].

Clinical decision-making is an essential part of the professional performance of medical staff, especially nurses and emergency medical technicians, which includes analyzing information, making decisions, and implementing these decisions in the clinical environment. Therefore, the awareness and appropriate performance of personnel in this regard can lead to correct clinical decision-making in critical situations.

Another variable examined in the present study was the examination of stressors in the study subjects. After the intervention, the mean score of stressors in the intervention group was lower than that in the control group. Several studies were consistent with the present results.

A study by Namdari et al. (2015) to investigate the level of job stress and its relationship with quality of life in employees of the Emergency Medical Incident Management Center in Kerman showed that there was a strong and negative relationship between quality of life and job stress, and reducing job stress can increase the quality of life of employees [30].

In a study conducted by Aghaei et al. (2019) with the aim of investigating the effectiveness of crisis management training based on an interprofessional approach on the ability of military nurses to deal with crises in selected military hospitals in Tehran. The findings indicated that crisis management training based on an interprofessional approach was effective in improving the ability of military nurses to deal with crises and could be optimally used in the educational planning of military organizations [31].

In a study conducted by Saeedi Mehr et al. (2014) with the aim of investigating the effect of crisis management training on nurses’ awareness of managing crisis situations, the results showed that nurses’ awareness of facing crises and how to deal with them increased after participating in the educational programs compared to before [32].

In a study conducted by Saberinia et al. (2012) with the aim of identifying stress factors that cause dissatisfaction in pre-hospital emergency staff in Kerman city, the main issues related to stress and dissatisfaction of staff are personal problems, organizational problems, inadequate coordination, and problems related to society. In general, the study showed that considering a psychotherapy team for staff and creating job rotation between sites are constructive suggestions of this study to improve staff performance and improve the emergency medical system in Kerman city [33].

In a study conducted by Ko et al. (2016) showed high stress levels and stressors among nurses working in oncology outpatient departments [34].

The study by Froutan et al. showed that stress management training can play an acceptable role in reducing the level of anxiety and increasing the resilience of emergency medical workers. Since reducing job stress in these individuals can lead to better clinical services, it is essential to use these effective strategies to reduce job stress and increase resilience [19]. A study by Mehni et al. showed that implementing a virtual stress management training program helps improve the general health of pre-hospital emergency personnel; therefore, its use is recommended as an effective method for empowering human resources [35].

While EMS personnel face high stress [14, 16, 20], few studies have tested how crisis training impacts their decision-making under pressure. Therefore, implementing more inclusive and responsive management to stressors can help reduce employee stress levels in crisis situations. The results showed that continuous education programs on stress management are highly recommended.

Based on the results, there was a statistically significant difference between the average score of employees’ job stress and their education and work experience. So that with increasing education and work experience, job stress increased. The existence of more job stress in employees with more work experience and older age can be explained by the fact that with increasing work experience and age of employees, the ability of individuals to adapt and tolerate stressful work conditions decreases and it is natural that job stress becomes more evident in them. On the other hand, with increasing work experience, the duration of employees’ presence in the Emergency Medical Services Center increases and they encounter more stress-causing factors. Regarding the increase in the level of education and the increase in job stress, it can also be stated that with the increase in the level of education, the process of doing the job becomes more specialized, which in itself can cause an increase in job stress. In a study by Namdari and colleagues, which was conducted to determine the level of job stress and examine its relationship with the quality of life in emergency medical services employees, they reached similar results [30]. However, in a study conducted by Khaghani Zadeh et al. to investigate job stress in healthcare workers, their results were not consistent with the present study, which could be due to the difference in the measurement tools of the study variables with the present study [36]. In a study by Ko et al. (2016) aimed to identify stress levels and stressors of nurses working in outpatient oncology departments. Demographic variables of age and nursing experience showed a positive and significant relationship with work-related stress scores. Although younger and less experienced nurses had lower mean stress scores than older and more experienced nurses, continuing education programs and tailored interventions would be beneficial for all oncology nursing staff [34].

The mean clinical decision-making score was also significantly associated with age, education, and work experience. Employees with more work experience and older age can be explained by the fact that as employees’ work experience and age increase, their ability to adapt and tolerate working conditions decreases, affecting their decision-making. Contrary to the results of the present study, in a study conducted by Imani et al. (2011) with the aim of determining the level of nurses’ awareness of crisis management and its related factors in Shahid Mohammadi Hospital, Bandar Abbas in 2009, The level of awareness of 3.2% of nurses regarding crisis management was very high, 16.6% was high, 52.3% was moderate, and 27.9% was low. In the study of statistical correlations, it was determined that there was a direct relationship between the level of education, type of work shift, participation in crisis maneuvers, and membership in the crisis committee with individuals’ awareness of crisis management [37].

The training program of the present study can be used as a guide for crisis management training, and it is also recommended training programs should prioritize simulation for younger, less experienced staff. Although these recommendations are specific to the Iranian EMS environment, they may have broader applicability to other developing EMS systems in resource-limited environments.

### Limitations

Despite its significant results, the present study also had limitations. This single-center study may not generalize to rural EMS with fewer training resources. Also, other limitations of the study included the small sample size, short follow-up, reliance on self-report, lack of generalizability.

It should be noted that this study reported large effect sizes, which may be overestimated due to factors such as self-report measures, short follow-up, and small sample size, and require cautious interpretation and replication in the future.

## Conclusion

The results showed that by implementing educational interventions, job stress and job strain can be prevented in emergency medical services personnel and lead to better management of stressors and improved clinical decision-making. Therefore, it is recommended that appropriate training programs be developed for this job group that is most closely related to crises. Also, considering the relationship of these variables with demographic factors such as education and work experience, training programs need to be designed according to these factors. This training model could reduce burnout in high-stress EMS settings. Therefore long-term studies should assess skill retention beyond 3 months.

## Data Availability

The datasets generated and/or analysed during the current study are not publicly available due [REASON WHY DATA ARE NOT PUBLIC] but are available from the corresponding author on reasonable request.
